# Integrin α_V_β_3_ can substitute for collagen‐binding β_1_‐integrins *in vivo* to maintain a homeostatic interstitial fluid pressure

**DOI:** 10.1113/EP086902

**Published:** 2018-04-30

**Authors:** Åsa Lidén, Tine Veronika Karlsen, Bengt Guss, Rolf K. Reed, Kristofer Rubin

**Affiliations:** ^1^ Department of Biomedicine University of Bergen Jonas Lies vei 91 N‐5009 Bergen Norway; ^2^ Department of Biomedical Sciences and Veterinary Public Health Swedish University of Agricultural Sciences Box 7036 SE‐750 07 Uppsala Sweden; ^3^ Centre for Cancer Biomarkers (CCBIO) University of Bergen Bergen Norway; ^4^ Department of Laboratory Medicine Translational Cancer Research Medicon Village Lund University SE‐223 63 Lund Sweden; ^5^ Department of Medical Biochemistry and Microbiology Science for Life laboratories Uppsala University BMC Box 582 SE 751 23 Uppsala Sweden

**Keywords:** contraction, extracellular matrix, microcirculation

## Abstract

**New Findings:**

- **What is the central question of this study?**
  Collagen‐binding β_1_‐integrins function physiologically in cellular control of dermal interstitial fluid pressure (*P*_IF_) *in vivo* and thereby participate in control of extravascular fluid volume. During anaphylaxis, simulated by injection of compound 48/80, integrin α_V_β_3_ takes over this physiological function. Here we addressed the question whether integrin α_V_β_3_ can replace collagen‐binding β_1_‐integrin to maintain a long‐term homeostatic *P*_IF_.
- **What is the main finding and its importance?**
  Mice lacking the collagen‐binding integrin α_11_β_1_ show a complex dermal phenotype with regard to the interstitial physiology apparent in the control of *P*_IF_. Notably dermal *P*_IF_ is not lowered with compound 48/80 in these animals. Our present data imply that integrin α_V_β_3_ is the likely candidate that has taken over the role of collagen‐binding β_1_‐integrins for maintaining a steady‐state homeostatic *P*_IF_. A better understanding of molecular processes involved in control of *P*_IF_ is instrumental for establishing novel treatment regimens for control of oedema formation in anaphylaxis and septic shock.

**Abstract:**

Accumulated data indicate that cell‐mediated contraction of reconstituted collagenous gels *in vitro* can serve as a model for cell‐mediated control of interstitial fluid pressure (*P*_IF_) *in vivo*. A central role for collagen‐binding β_1_‐integrins in both processes has been established. Furthermore, integrin α_V_β_3_ takes over the role of collagen‐binding β_1_‐integrins in mediating contraction after perturbations of collagen‐binding β_1_‐integrins *in vitro*. Integrin α_V_β_3_ is also instrumental for normalization of dermal *P*_IF_ that has been lowered due to mast cell degranulation with compound 48/80 (C48/80) *in vivo*. Here we demonstrate a role of integrin α_V_β_3_ in maintaining a long term homeostatic dermal *P*_IF_ in mice lacking the collagen‐binding integrin  α_11_β_1_ (α11^−/−^ mice). Measurements of *P*_IF_ were performed after circulatory arrest. Furthermore, cell‐mediated integrin α_V_β_3_‐directed contraction of collagenous gels *in vitro* depends on free access to a collagen site known to bind several extracellular matrix (ECM) proteins that form substrates for α_V_β_3_‐directed cell attachment, such as fibronectin and fibrin. A streptococcal collagen‐binding protein, CNE, specifically binds to and blocks this site on the collagen triple helix. Here we show that whereas CNE perturbed α_V_β_3_‐directed and platelet‐derived growth factor BB‐induced normalization of dermal *P*_IF_ after C48/80, it did not affect α_V_β_3_‐dependent maintenance of a homeostatic dermal *P*_IF_. These data imply that dynamic modification of the ECM structure is needed during acute patho‐physiological modulations of *P*_IF_ but not for long‐term maintenance of a homeostatic *P*_IF_. Our data thus show that collagen‐binding β_1_‐integrins, integrin α_V_β_3_ and ECM structure are potential targets for novel therapy aimed at modulating oedema formation and hypovolemic shock during anaphylaxis.

## 1 INTRODUCTION

Loose connective tissue structures surround all peripheral blood and lymph vessels, nerves and muscles, as well as underlying epithelial sheets forming what is commonly referred to as the interstitium. The interstitium harbours the extracellular fluid, whose volume amounts to some 15% of the total body weight. Interstitial fluid volume is determined by the influx of fluid across the capillary wall and drainage via the lymphatics. Capillary filtration is determined by the colloidal osmotic pressures across the capillary wall and the capillary pressure that is determined from the myogenic activity of the smooth muscle in the microvasculature and the permeability of the microvascular barrier (Curry & Adamson, 2013; Michel & Curry, 1999). The interstitial volume is the volume resulting from the balance between this influx of fluid and the lymphatic drainage. Finally, the interstitial fluid pressure (*P*_IF_) is a function of the interstitial fluid volume and the interstitial compliance, but as we have shown, it is also actively controlled by connective tissue cells. In skin *P*_IF_ is normally slightly below ambient pressure, i.e. around −1 mmHg compared at a capillary hydostatic pressure of around 10 mmHg and a net capillary pressure, i.e. the net pressure that creates filtration across the capillaries, of 0.5–1 mmHg (Reed, Liden, & Rubin, 2010). *P*_IF_ normally acts to maintain a constant interstitial volume while in particular conditions like inflammation a lowered *P*_IF_ transiently becomes the main driving force for the rapid initial fluid movement out of the microvasculature during early innate immunity responses (Reed et al., 2010). A lowering of *P*_IF_ by even a few mmHg will represent an important part of the driving force for capillary filtration together with increased capillary hydrostatic pressure and increased capillary permeability since the lowering of *P*_IF_ must be compared with a net capillary filtration pressure of a 0.5–1 mmHg (Reed et al., 2010). Once oedema has formed, *P*_IF_ will reach positive values and further maintenance of filtration and oedema relies on increased capillary hydrostatic pressure and increased capillary permeability.

Under steady‐state conditions connective tissue cells balance the slightly negative *P*_IF_ by exerting tensional forces that maintain the proteoglycan/hyaluronan ground substance of the extracellular matrix (ECM) in an underhydrated state (Reed et al., 2010). The necessary force is generated by the cytoskeletal machinery that connects to ECM fibres via integrins (Berg, Rubin, & Reed, 2001; Reed, Rubin, Wiig, & Rodt, 1992). At homeostasis β_1_‐integrins are operative in rat and mouse dermis whereas during inflammatory reactions, in which  *P*_IF_ is lowered, e.g. during anaphylaxis, there is a shift in integrin usage such that the α_V_β_3_‐integrin, and not β_1_‐integrins, connects the cellular contractile apparatus to ECM fibres (Liden, Berg, Nedrebø, Reed, & Rubin, 2006; Svendsen, Liden, Nedrebø, Rubin, & Reed, 2008). Available data suggest that the collagen‐binding integrins α_2_β_1_ (Rodt, Åhlen, Berg, Rubin, & Reed, 1996) and α_11_β_1_ (Svendsen et al., 2009) are operative to maintain a homeostatic *P*_IF_ in rat and mice dermis, respectively. In α_11_β_1_‐deficient mice blockage of β_1_‐integrins does not lower *P*_IF_ whereas such blockage lowers *P*_IF_ in wild‐type mice (Reed et al., 1992; Svendsen et al., 2009). Local administration of platelet‐derived growth factor (PDGF)‐BB normalizes *P*_IF_ in mouse and rat dermis in which *P*_IF_ has been lowered by mast cell degranulation (Liden et al., 2006; Rodt et al., 1996). This effect of PDGF‐BB requires functional integrin α_V_β_3_ (Liden et al., 2006). Furthermore, dermal *P*_IF_ is not significantly lowered in α_11_β_1_‐deficient mice, but readily lowered in wild‐type mice during compound 48/80 (C48/80)‐induced anaphylaxis (Svendsen et al., 2009).

The traits for integrin usage in cellular control of *P*_IF_ *in vivo* are paralleled by cell‐mediated contraction of three‐dimensional reconstituted collagen gels *in vitro*. Thus, collagen‐binding β_1_ integrins mediate, when present, the cell–collagen contacts that are necessary for contraction (Gullberg et al., 1990); in their absence integrin α_V_β_3_ becomes operative (Grundström Grundström, Mosher, Sakai, & Rubin, 2003). Integrin α_V_β_3_‐directed contraction by myoblasts requires that the cells synthesize fibronectin, a synthesis that in these cells is stimulated by PDGF‐BB (Liden et al., 2008; van Wieringen et al., 2010). Available data suggest that fibronectin forms a bridge between the collagen fibres and integrin α_V_β_3_ thereby enabling collagen gel contraction (Liden et al., 2008; van Wieringen et al., 2010). Fibronectin binds collagen monomers at a discrete collagen site that also binds collagenases, discoidin domain receptor 2, fibromodulin and fibrinogen (Farndale et al., 2008; Fields, 2014; Howes et al., 2014; Kalamajski, Bihan, Bonna, Rubin, & Farndale, 2016; Manka et al., 2012; Reyhani et al., 2014; van Wieringen et al., 2010). This site is also recognized by the collagen‐binding streptococcal protein CNE, which inhibits α_V_β_3_‐directed, fibrin‐ or fibronectin‐dependent collagen gel contraction by myoblasts (Reyhani et al., 2014; van Wieringen et al., 2010).

Here we investigated the role of integrin α_V_β_3_‐integrin in maintaining *P*_IF_ in the dermis of mice with a constitutively perturbed function of collagen‐binding β_1_‐integrins, such as in α_11_β_1_‐deficient mice (Svendsen et al., 2009). Furthermore, we investigated the potential role of collagen‐binding proteins that may bridge the collagen fibres to cellular α_V_β_3_ by investigating potential effects of the streptococcal protein CNE on cellular control of *P*_IF_.

## 2 METHODS

### 2.1 Ethical approval

The animal experiments were conducted according to the European Convention for the Protection of Vertebrates Used for Scientific Purposes, Norway and were approved by the Institutional Committee at University of Bergen and the Norwegian Animal Research Authority (permission numbers 2006007 and 2006006). The investigators understand the ethical principles under which the journal operates. The study reported here complies with these animal ethics. The mice were housed at the animal facility at Faculty of Medicine and experiments performed at the Department of Biomedicine. The mice had free access to food and water and were kept under a 12 h–12 h day–night cycle.

Two strains of mice were used in the study. The α_11_^−/−^ mice were in a C57BL/6 background (Popova et al., 2007) were a kind gift from professor D. Gullberg, Department of Biomedicine, University of Bergen. For the C48/80 study, BALB/c mice were used in accordance with previous studies using the mast cell degranulating agent C48/80 (Liden et al., 2006). The origin of the BALB/c mice stock is detailed in Liden et al. (2006) and the mice have been bred and maintained at University of Bergen. Anaesthesia was induced with a mixture of ketamine (12.2 mg ml^−1^; Ketalar, Pfizer, New York, USA) and medetomidine (24.3 μg ml^−1^; Domitor, Orion Pharma, Espoo, Finland) in saline injected intramuscularly (0.1 ml per 10 g body weight). Surgical procedures involved administration of an intravenous catheter in the external jugular vein in Groups B and C (see below). Measurements of interstitial fluid pressure (*P*_IF_) were performed on the dorsal side of the hind paw with the mouse lying on its back. After a control measurement with intact circulation, the remaining measurements (90 min) were performed after circulatory arrest and the animal was killed with cervical dislocation in Group A (see below). In Groups B and C (see below) the animals were killed with intravenous saturated KCl. Furthermore, the duration of anaesthesia in all three groups was no more than 5–10 min including measurement of control *P*_IF_ and i.v. injections in any of the groups. Before and during the experiments sufficient depth of anaesthesia was confirmed by lack of response to hindlimb toe pinch.

### 2.2 Reagents

Purified NA/LE Hamster Anti‐Mouse CD61 IgG_1_ that blocks α_V_β_3_‐integrin‐mediated cell adhesion was obtained from BD Biosciences (San Jose, CA, USA). The streptococcal protein CNE was produced and purified as described earlier (Lannergård, Frykberg, & Guss, 2003). C48/80 was obtained from Sigma‐Aldrich (St. Louis, MO, USA).

### 2.3 Interstitial fluid pressure

*P*_IF_ was measured by the micropuncture technique (Svendsen et al., 2009). Briefly, sharpened glass microcapillaries with tip diameter 4–7 μm were filled with 0.5 m saline and connected to a servocontrolled counterpressure system. A measurement was accepted when (1) there was no stretch or indentation in the skin from the pipette at the site of the puncture; (2) gain on the servo‐controlled system could be changed without altering the pressure recording (e.g. there was free communication for fluid across the pipette tip) and (3) recording of zero (ambient) pressure in a saline cup at the level of puncture did not change from before to after the measurement. Zero was taken as ambient pressure recorded in a saline filled cup at the level of measurement.

## 3 EXPERIMENTAL GROUPS

### 3.1 Effects of the anti‐integrin β_3_ IgG on dermal interstitial fluid pressure

After measurement of control *P*_IF_ with intact circulation, circulatory arrest was induced by dislocation of the neck. Thereafter 1 μl of anti‐integrin β_3_ IgG (1 μg μl^−1^) was injected intradermally and *P*_IF_ was measured for the next 90 min. Measurements were performed in wild‐type C57BL/6 mice and in littermate mice deficient in α_11_β_1_.

### 3.2 Effects of compound 48/80 and subsequent injection of PDGF‐BB alone or with CNE

After a control measurement of *P*_IF_ the mice were injected intravenously with 200 μg C48/80 in 100 μl phosphate‐buffered saline. C48/80 induces a generalized mast cell degranulation that as part of the clinical picture is associated with a lowering of *P*_IF_ within 30 min. Also, the effect is seen as increased respiratory rate and lowering of blood pressure. Circulatory arrest was induced by i.v. injection of saturated KCl 2 min after injection of C48/80. Measurement of *P*_IF_ was started and continued for the next 90 min. Mice that did not demonstrate a lowering of *P*_IF_ of at least 0.5 mmHg were excluded from the study since a lack of response to C48/80 means that PDGF‐BB will not have a lowered *P*_IF_ to act on. One microlitre of PDGF‐BB (0.7 μg ml^−1^) was injected intra‐dermally after 30 min either alone or combined with CNE at 0.7 mg ml^−1^.

### 3.3 Effects of CΝΕ in wild‐type and α_11_β_1_‐deficient mice

After control measurement of *P*_IF_ with intact circulation, the animals were given saturated KCl intravenously to induce circulatory arrest. One microlitre of CNE at 0.7 μg ml^−1^ was injected subcutaneously and measurement of *P*_IF_ continued for 90 min.

### 3.4 Statistical methods

Data are presented as means ± SD unless specified otherwise. Repeated measurements ANOVA and *post‐hoc* test (Sidak's multiple comparison test) correcting for multiple corrects were used. Measurements of *P*_IF_ were compared using one‐ and two‐tailed Student's *t* test as specified in Results. *P* < 0.05 was considered statistically significant.

## 4 RESULTS

### 4.1 Effects of anti‐integrin β_3_ IgG on dermal interstitial fluid pressure

**Figure 1:**
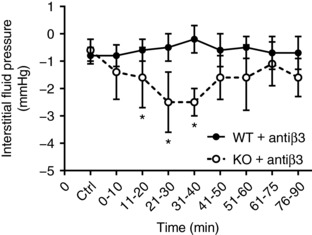
Interstitial fluid pressure in wild‐type (WT; α11^+/+^) mice (filled circles, *n* = 8) and knockout (KO; α11^−/−^) mice (open circles, *n* = 8). Administration of 1 μl anti‐integrin β_3_ IgG (1 μg μl^−1^) resulted in a significant lowering of interstitial fluid pressure in KO (α11^−/−^) mice. Values are means ± SD; *P < 0.05

In accordance with previously reported findings showing that β_1_‐integrin and not α_V_β_3_ is operative in maintaining *P*_IF_ at homeostasis, local intradermal injection of anti‐integrin β_3_ IgG in wild‐type naïve C57BL/6 mice had no effect on *P*_IF_ (Figure 1). In contrast, intradermal injection of the IgG in α_11_β_1_‐deficient mice (α_11_^−/−^ mice) resulted in a lowering of *P*_IF_ from control values down to between −2 and −2.5 mmHg (21–40 min after injection, a significant lowering when compared to α^+/+^ mice at this time point (*P* < 0.0001, two‐tailed *post hoc t* test)

### 4.2 Effects of compound 48/80 and subsequent injection of PDGF‐BB alone or with CNE

**Figure 2:**
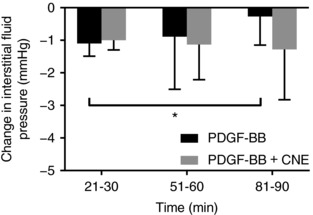
C48/80 was used to lower interstitial fluid pressure. Subsequent injection of 1 μl of platelet‐derived growth‐factor BB (PDGF‐BB) (0.7 μg ml^−1^) (black bars, *n* = 7) resulted in a significant attenuation of the lowered interstitial pressure back towards the level prior to C48/80. Injection of PDGF‐BB as above together with the streptococcal protein CNE (0.7 mg ml^−1^) (grey bars, *n* = 8) attenuated the effect of PDGF and interstitial pressure did not change from the lowered value back towards the level priori to C48/80. Values are means ± SD; *P < 0.05

Intravenous injection of C48/80 resulted in a significant lowering of dermal *P*_IF_ compared to control in BALB/c mice (Figure 2) (*P* < 0.001 when using paired comparison and two‐tailed *t* test). Subsequent injection of 1 μl PDGF‐BB returned *P*_IF_ to control values (Figure 2) while PDGF‐BB injected concomitant with CNE did not change *P*_IF_ from its lowered value. *P*_IF_ recorded 21–30 min after injection of C48/80 was not significantly different from the value at 81–90 min when CNE was injected together with PDGF‐BB (*P* = 0.684 with paired comparison and two‐tailed *t* test) and significantly lower than its own control value recorded prior to the injection of C48/80 at 51–60 min (*P* = 0.03) and at 81–90 min (*P* = 0.06) using a two‐tailed *t* test and paired comparison). This effect of CNE cannot be attributed to interference with PDGF‐BB signalling since CNE does not inhibit PDGF‐BB‐elicited phosphorylation of PDGF receptors in cultured cells (Supplementary Figure 2B in van Wieringen et al., 2010). When PDGF‐BB was injected alone, *P*_IF_ returned towards control and *P*_IF_ at 81–90 min was significantly different from *P*_IF_ at 21–30 min (*P* = 0.03) but not from its own control (*P* = 0.72) measured prior to injection of C48/80 (in both cases using paired comparison and two‐tailed testing).

### 4.3 Effects of CNE in wild‐type and α_11_β_1_‐deficient mice

**Figure 3:**
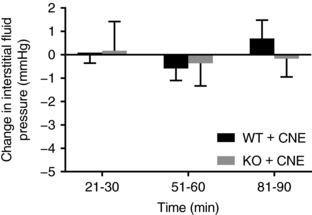
Effect of subcutaneous injection of 1 μl of CNE at 0.7 μg ml^−1^ on interstitial fluid pressure in 7 wild‐type (WT; α11^+/+^) mice (black bars) and 7 knockout (KO; α11^−/−^) mice (grey bars). Data are shown as changes from control in interstitial fluid pressure. The control interstitial fluid pressure, measured prior to the injection of CNE, was −0.7 ± 0.4 mmHg (*n* = 7) in wild‐type and −1.4 ± 0.4 mmHg (*n* = 7) in ΚΟ (α11^−/−^) mice. One‐way repeated ANOVA showed no significant effects of CNE on interstitial fluid pressure in any of the two genotypes. Values are means ± SD

Injection of 1 μl 0.7 mg ml^−1^ CΝΕ in wild‐type and α_11_β_1_‐deficient (α_11_^−/−^) mice did not change *P*_IF_ compared to the respective controls (Figure 3). *P*_IF_ in both wild‐type and α_11_β_1_‐deficient mice was unaffected by injection of CNE (*P* > 0.05 using one‐way repeated ANOVA). *P*_IF_ in the α_11_β_1_‐deficient mice was lower in this experimental series than in wild‐type. The control *P*_IF_ values did not, however, differ between wild‐type and α_11_β_1_‐deficient mice in the experimental series shown in Figure 1, nor in those reported by Svendsen et al. (2009).

## 5 DISCUSSION

Here we show that the integrin α_V_β_3_ functions physiologically to maintain the homeostatic *P*_IF_ in mouse dermis lacking the integrin α_11_β_1_. During acute inflammatory reactions collagen‐binding β_1_‐integrins decouple and their role in controlling *P*_IF_ is taken over by the α_V_β_3_ integrin (Liden et al., 2006; Svendsen et al., 2008). Our present data are a further elaboration on how *P*_IF_ can be modulated by cellular and molecular pathways and show that the α_V_β_3_ integrin can participate in *P*_IF_ control also in the absence of inflammation. The data also expand on previous findings on a potential role of the α_11_β_1_ integrin in control of dermal *P*_IF_ in mice (Svendsen et al., 2009). Taken together with the data presented here, it is possible to conclude that the collagen‐binding β_1_‐integrin α_11_β_1_ is a key operator in maintaining a homeostatic *P*_IF_ in normal dermis. In mouse dermis lacking α_11_β_1_ (α_11_^−/−^ mice), *P*_IF_ was only marginally lowered after induction of anaphylaxis by the mast cell degranulator C48/80 (Svendsen et al., 2009) suggesting that α_V_β_3_ integrin‐operated *P*_IF_ control works also during anaphylaxis, which is in line with previously published reports (Liden et al., 2006; Svendsen et al., 2008).

In a previous publication, we presented data on a role of the collagen‐binding β_1_‐integrin α_2_β_1_ in controlling *P*_IF_ in rat dermis (Rodt et al., 1996). This conclusion was based on experiments in which the anti‐rat α_2_β_1_ monoclonal antibody Ha1/29 (Mendrick & Kelly, 1993) lowered *P*_IF_ in naïve rat dermis. It is thus possible that mice and rats differ as to preferred usage of collagen‐binding β_1_‐integrin to control dermal *P*_IF_. Alternatively, both integrins are required and perturbation of any of them distorts dermal *P*_IF_‐control. It can furthermore not be excluded that the Ha1/29 antibody inhibits both α_2_β_1_ and α_11_β_1_. It is not clear whether collagen‐binding β_1_‐integrins bind directly to collagen molecules in the ECM fibres *in vivo* or only via accessory proteins as has been suggested to be the case for chondrocyte binding to cartilage collagenous fibres (Woltersdorf et al., 2017). Our present data do not discriminate between these two possibilities but together with previously reported data show that integrins play an important physiological role in controlling *P*_IF_.

To further delineate α_V_β_3_ integrin‐operated *P*_IF_ control in mouse dermis deficient in the α_11_β_1_ integrin (α_11_^−/−^ mice), we took advantage of the streptococcal protein CNE. CNE binds to and blocks a collagen site that is necessary for binding of several proteins that can function as a bridge between cellular α_V_β_3_ and the collagen fibres, such as fibrin and fibronectin. Integrin α_V_β_3_‐mediated contraction of collagen gels *in vitro* relies on these interactions and is inhibited by CNE (Reyhani et al., 2014; van Wieringen et al., 2010). Our present data demonstrate an *in vivo* effect of CNE, namely that it inhibited PDGF BB‐induced and integrin α_V_β_3_‐mediated normalization of *P*_IF_ that has been lowered by induction of anaphylaxis in naïve mouse dermis using the mast cell degranulator C48/80. This implies, first, that the ECM is altered during early innate immune responses. Second, that a collagen‐binding site needs to be available in order for the cellular binding to ECM fibres via α_V_β_3_ to occur, a defined site known to bind several proteins that can associate with collagen fibres (Farndale et al., 2008; Fields, 2014; Howes et al., 2014; Kalamajski et al., 2016; Manka et al., 2012; Reyhani et al., 2014; van Wieringen et al., 2010). Based on our present finding that CNE had no effect on *P*_IF_ in naïve mouse dermis lacking α_11_β_1_ (α_11_^−/−^ mice) or in wild‐type dermis it can be concluded that integrin α_V_β_3_‐directed processes that are operative in *P*_IF_ control during homeostasis differ from the dynamic changes resulting from acute inflammatory reactions. Based on the induction of an acute inflammation in mouse dermis lacking α_11_β_1_ (α_11_^−/−^ mice) not resulting in a lowering of *P*_IF_, the present findings with CNE suggest the need for a change of ECM build‐up in order for the tissue to be able to respond to inflammatory insults by forming oedema.

In conclusion, the present data show that integrin α_V_β_3_ can fully substitute for loss of collagen‐binding β_1_‐integrins with regard to maintaining a homeostatic dermal *P*_IF._ Taken together with results presented by Svendsen et al. (2009), the data also imply that α_V_β_3_ integrin‐operated *P*_IF_ control does not respond to acute inflammatory challenges and thereby does not enable oedema formation during innate immunity responses. Furthermore, our data show that whereas in normal dermis α_V_β_3_ integrin‐operated *P*_IF_ control requires changes of the ECM build‐up, they are not needed in dermis in which impaired collagen‐binding β_1_‐integrin activity is a constitutive property.

## AUTHOR CONTRIBUTIONS

The experiments were performed in the laboratory space of the Cardiovascular Research Group at Department of Biomedicine, University of Bergen. CNE was prepared at the Swedish University of Agricultural Sciences, Uppsala, Sweden.  K.R. and R.K.R. designed the study and wrote the manuscript. T.V.K. and Å.L. performed the experiments. B.G. prepared and quality assured CNE. K.R., R.K.R., T.V.K. and Å.L. analysed the data. All contributors participated in the writing of the manuscript. All authors approved the final version of the manuscript and agreed to be accountable for all aspects of the work in ensuring that questions related to the accuracy or integrity of any part of the work are appropriately investigated and resolved. All persons designated as authors qualify for authorship, and all those who qualify for authorship are listed.
